# The Safety and Efficacy of Microporous Polysaccharide Hemospheres in Terms of the Complication Rates in Total Hip Arthroplasty for Femoral Neck Fractures: A Control-Matched Retrospective Cohort

**DOI:** 10.3390/life14020177

**Published:** 2024-01-25

**Authors:** Olga Pidgaiska, Marcel Niemann, Karl Braun, Andrej Trampuz, Stavros Goumenos, Ulrich Stöckle, Sebastian Meller

**Affiliations:** 1 Charité-Universitätsmedizin Berlin, Corporate Member of Freie Universität Berlin, Humboldt-Universität zu Berlin and Berlin Institute of Health, Center for Musculoskeletal Surgery (CMSC), Augustenburger Platz 1, 13353 Berlin, Germany; olga.pidgaiska@charite.de (O.P.); marcel.niemann@charite.de (M.N.); andrej.trampuz@charite.de (A.T.); stavros.goumenos@charite.de (S.G.); ulrich.stoeckle@charite.de (U.S.); 2 Sytenko Institute of Spine and Joint Pathology, Academy of Medical Science, Ukraine, Pushkinska Str. 80, 61024 Charkiw, Ukraine; 3 Department of Trauma Surgery, Klinikum Rechts der Isar, Technical University of Munich, Ismaninger Str. 22, 81675 Munich, Germany; karl.braun@tum.de

**Keywords:** microporous polysaccharide hemospheres, postoperative haematoma, periprosthetic joint infection, complications after total hip arthroplasty

## Abstract

Aims. This study aimed to assess the safety and efficacy of microporous polysaccharide hemospheres (MPSHs) in managing blood loss and reducing the risk of postoperative haematoma and early periprosthetic joint infection (PJI) following total hip arthroplasty (THA) for femoral neck fracture (FNF), in the context of the existing treatment challenges. Methods. A control-matched retrospective analysis of 163 patients undergoing unilateral primary THA for displaced FNF between 2020 and 2023 was performed. The study group consisted of 74 patients who received MPSH administered intraoperatively. The control group consisted of 89 patients who received no topical haemostatics. One-to-one case–control matching between groups was performed. The primary outcome was a perioperative change in the haematologic values (haemoglobin, red blood cell count, haematocrit, platelet concentration) and transfusion rate. The secondary outcomes were the incidence of postoperative local haematoma formation, prolonged wound secretion, surgical site infection (SSI), and PJI within 3 months of surgery. Results. Our analysis found no statistically significant differences in the haematologic parameters between the control and study cohorts. The changes in the haemoglobin concentration were not significant between the control group (3.18 ± 1.0 g/dL) and the treatment group (2.87 ± 1.15 g/dL) (*p* = 0.3). There were no significant differences (*p* = 0.24) in the haematocrit and red blood cell concentration (*p* = 0.15). The platelet levels did not significantly differ (*p* = 0.12) between the groups. Additionally, we found no significant discrepancy in the incidence of early PJI or blood transfusion rates between the groups. No adverse effects following MPSH use were recorded in the study group. Conclusions. Routine use of MPSH in THA for FNF management appears to be safe, with no observed adverse events related to Arista^®^ use. Although there was a tendency towards reduced blood loss in the Arista^®^ AH group, MPSH did not significantly impact bleeding complications, local haematoma formation, or subsequent PJI.

## 1. Background

The demand for total hip arthroplasty (THA) following femoral neck fracture (FNF) is on the rise, particularly among elderly patients, who necessitate urgent medical care and surgical intervention [1,2]. Recent studies have consistently demonstrated that THA offers superior outcomes and enhanced cost-effectiveness in comparison to osteosynthesis or hip hemiarthroplasty, especially in older patients with high functional demands [3,4,5,6]. Given these findings, THA is increasingly being recognised as the preferred treatment modality for patients with high demands, offering significant benefits in terms of patient recovery and long-term prognoses.

Although total hip arthroplasty (THA) is recognised as a highly successful intervention in treating femoral neck fracture (FNF), it is imperative to consider the potential complications associated with this procedure. Multiple studies have demonstrated higher readmission rates due to complications following total hip arthroplasty for femoral neck fracture compared to THA performed due to osteoarthritis, one of them being an increased incidence of postoperative haematoma formation (1% to 10%) [7,8,9]. Various risk factors have been associated with an increased risk of haematoma formation after THA, including patient age, comorbidities, anticoagulant therapy, the duration of surgery, a higher ASA score, a high BMI, and hormonal therapy [3,10,11,12,13,14].

Around 30–40% of elderly patients who sustain a hip fracture are estimated to be on some sort of anticoagulation or antiplatelet therapy due to pre-existing medical conditions [15,16]. Additionally, it is noteworthy that between 2 and 20% of patients undergoing surgery for hip fractures are administered direct oral anticoagulants [17,18]. These substances are known to significantly increase the risk of postoperative bleeding, including haematoma formation [19,20,21,22], leading to poor clinical outcomes and higher mortality rates [8]. A recently published study employing multivariate analysis revealed that haematoma formation is an independent risk factor leading to poorer outcomes in patients undergoing surgery [8]. Specifically, this includes lower functional results, increased complication rates, and higher mortality. Due to increased wound tension and subsequent reduced tissue perfusion, these haematomas can hinder wound healing but also increase the risk of surgical site infections (SSIs) and periprosthetic joint infections (PJIs) from 1–2% in patients undergoing elective THA to 7.3% in patients undergoing THA for FNF [20,21,23]. 

Tranexamic acid (TXA), an antifibrinolytic drug, is commonly used in arthroplasty surgery [24], spinal surgery [25,26], and orthopaedic oncology [27,28]. It has been repeatedly demonstrated to lower blood loss and complications. In addition, topical haemostatics are used but are less well studied. 

To reduce the risk of hematoma formation, excessive blood loss, and the need for transfusion, efficient coagulation management is vital in THA for FNF. Arista^®^ AH is a plant-based, absorbable surgical haemostatic powder that has emerged as a haemostatic agent in various surgical specialties, including cardiothoracic surgery, urology, and plastic surgery [29,30,31,32]. The proposed mechanism of action of the microporous polysaccharide hemospheres (MPSHs) of Arista^®^ AH is in expediting blood clot formation and generating a haemostatic plug. This appears to be promising for achieving rapid and effective haemostasis. However, there is a limited number of clinical studies appraising its efficacy and safety in orthopaedic and trauma surgery in primary hip and knee osteoarthritis [33,34,35].

This study assesses the effect of the use of MPSHs on bleeding-related complications and potential adverse events (AEs) in THA performed for FNF. The primary outcome was the perioperative change in the haematological values (haemoglobin, red blood cell count, haematocrit, platelet concentration) and transfusion rate. The secondary outcomes were the incidence of postoperative local hematoma, prolonged wound secretion, SSI, and PJI within three months following surgery.

## 2. Materials and Methods

This was a retrospective control-matched study of patients undergoing unilateral primary THA after FNF (Garden type III or IV) between January 2020 and March 2023 at a single study centre. All patients were routinely followed up for at least three months postoperatively. The study was conducted following the provisions of the Declaration of Helsinki. The local ethics committee approved the study protocol in advance (application number EA4/040/14). 

Inclusion criteria: patients with displaced femoral neck fractures (Garden III, IV) eligible for THA based on functional demand, scheduled for unilateral primary total hip replacement and eligible for a follow-up period three months postoperatively. The exclusion criteria included (1) an age younger than 18 years; (2) any history of surgery on the affected hip joint, which would potentially lead to a prolonged surgery duration and increased intraoperative blood loss; (3) pathological FNF; (4) taking direct anticoagulants, warfarin, or clopidogrel; (5) a history of liver disease, chronic renal insufficiency, or coagulopathy; and (6) postoperative anticoagulation with agents other than low-molecular-weight heparins.

**Figure 1:**
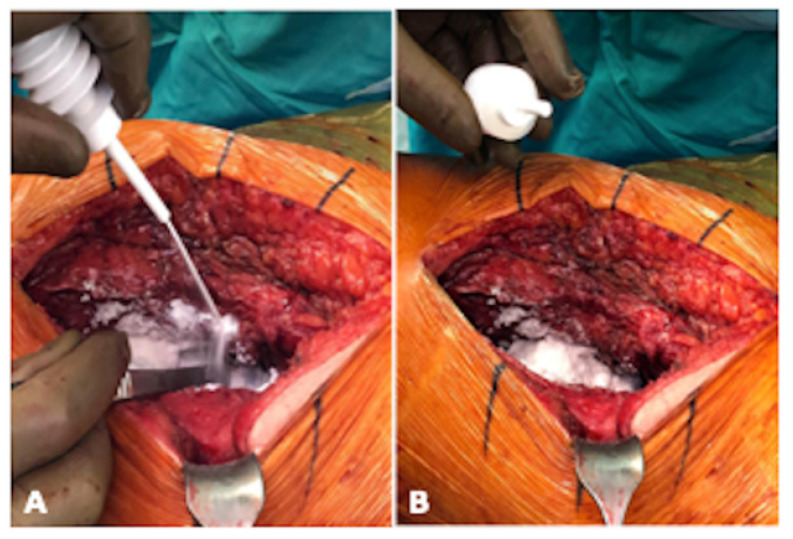
Intraoperative application of Arista^®^ AH during THA implantation in lateral position of the patient. (**A**) illustrates the intraoperative application of Arista^®^ AH periarticular and (**B**) shows the view of the periarticular soft tissue after application (photograph by Sebastian Meller).

During the study period, Arista^®^ AH was introduced as a locally applied agent during THA surgery. All patients followed a strict surgical protocol: firstly, patients underwent a comprehensive preoperative assessment. A weight-based single dose of antibiotics was intravenously administered within one hour prior to skin incision. All the THAs were performed using an anterolateral or lateral approach, and no drains were placed during surgery. General anaesthesia was administered during surgery. In the study group, 3 g of Arista^®^ AH was applied locally after THA implantation and before the closure of the fascia (Figure 1). All patients received an intravenous tranexamic acid bolus of 1 g at the beginning of surgery, to which we added 3 g of tranexamic acid injected topically into the hip joint after fascia closure. All patients received postoperative thrombosis prevention therapy using low-molecular-weight heparin (LMWH) (subcutaneous enoxaparin natrium 40 mg) for 14 days. Blood levels were measured in the morning of the first three postoperative days. Partial weight-bearing on the affected leg was permitted and a standard physiotherapy protocol was routinely applied. Postoperative X-rays were taken in two standard views. The minimum follow-up duration of this study was three months, during which patients attended follow-up visits where complaints and complications were recorded. All the THAs evaluated in this study were performed by two experienced orthopaedic surgeons. 

The demographic and clinical data were extracted using the study centre’s electronic medical database SAP (SAP ERP 6.0 EHP4, SAP AG, Walldorf, Germany). These included age, gender, body mass index (BMI), American Society of Anesthesiologists (ASA) grade, laboratory data, the blood products transfused, surgical complications (wound healing disorder, prolonged wound secretion more than seven days, reoperation), and thromboembolic complications following the THA implantation surgery.

The primary outcome assessed was the perioperative change in the haematologic values (haemoglobin, red blood cell count, haematocrit, platelet concentration) and transfusion rate. The secondary outcomes were evaluating the incidence of postoperative local haematoma, prolonged wound secretion, SSI, and PJI within three months following surgery. PJI was diagnosed using the EBJIS criteria by our interdisciplinary team [36]. The institutional protocol set the red blood cell transfusion threshold to a haemoglobin level of below 7.0 g/dL. Laboratory measures were assessed before surgery and at one timepoint within the first 72 h following surgery. 

For the statistical analysis, a 1:1 case–control matching was performed on patients treated with Arista^®^ AH (study group) and patients not treated with Arista^®^ AH (control group). The matching was based on age, gender, ASA score, and BMI, with tolerance limits set at five for age, one for ASA score, and three for BMI. Gender was matched precisely. The case–control matching was performed using SPSS (SPSS Statistics, Version 28, version 28.0.1.0 [142], IBM Corp., Armonk, NY, USA).

Subsequent statistical analyses were conducted using GraphPad Prism (GraphPad Prism 10, version 10.0.2 [232], GraphPad Holdings, LLC, San Diego, CA, USA). The Wilcoxon signed-rank test was used for dependent samples unless otherwise specified. Categorical variables were presented as numbers (percentage of the whole [%]), while continuous variables were expressed as mean ± SD (95% CI). All tests were two-tailed and the statistical significance was set at *p* ≤ 0.05.

## 3. Results

### 3.1. Demographic Data

A total of 163 patients met the inclusion criteria and were included in this study. There were 89 patients in the control group and 74 in the study group. In the control group, the patients ranged in age between 38 and 89 years, with an average age of 68. Among these, 38 (42.7%) were men and 51 (57.3%) were women. In the study group, the 74 patients were aged between 18 and 86 years, with an average age of 67. Among them, 30 (40.5%) were men and 44 (59.5%) were women. There were no significant differences in age (*p* = 0.637) and sex distribution (*p* = 0.781) between the groups. 

### 3.2. Case–Control Matched Comparisons

**Figure 2:**
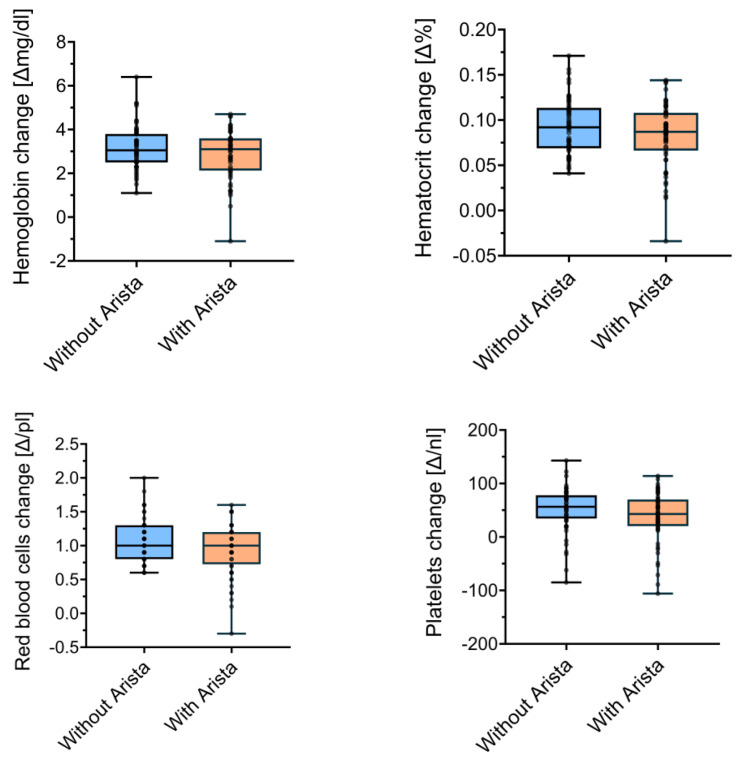
Perioperative changes in laboratory measures between groups in the matched analysis.

After patient matching, 56 patients per group were compared. The mean changes in Hb concentration did not differ (*p* = 0.3) between the control group (3.18 ± 1.0 g/dL) and the treatment group (2.87 ± 1.15 g/dL). There were no significant differences (*p* = 0.24) in Ht when comparing the control group (0.09 ± 0.03%) and the study group (0.08 ± 0.04%). The perioperative changes in the red blood cell concentration also did not significantly differ (*p* = 0.15) between the control (1.06 ± 0.32 g/dL) and the study group (0.93 ± 0.38 g/dL). Lastly, the platelet levels did not significantly differ (*p* = 0.12) between the control (51.38 ± 41.6 × 10^9^/L) and the study group (37.55 ± 47.67 × 10^9^/L). Figure 2 visualises these comparisons between the matched control and study groups.

### 3.3. Complications and Adverse Events

**Figure 3:**
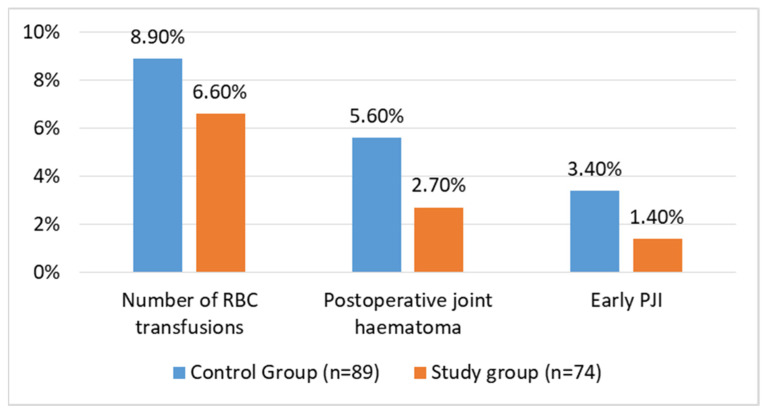
Complication rates after THA for FNF in matched groups of patients.

Early postoperative infections occurred in patients in both groups. In the control and study groups, three (3.4%) patients and one (1.4%) patient had an early PJI, respectively, and were treated with debridement, antibiotics, and implant retention (DAIR). In all instances, the DAIR procedure involved replacing the polyethylene liner and the head of the implant. The difference between the groups was not statistically significant (*p* = 0.407). Five patients in the control group (5.6%) and two in the study group (2.7%) developed a local haematoma during the follow-up period. This difference was not significant between the groups (*p* = 0.36). Three patients in the control group (3.4%) and one in the study group (1.4%) were treated surgically with the DAIR procedure. Lastly, there was no difference (*p* = 0.60) in the transfusion rates between the control (8 patients; 8.9%) and study groups (5 patients; 6.6%). These data are visualised in Figure 3.

## 4. Discussion

Our study aimed to evaluate the effectiveness and safety of routinely employing MPSHs in the haemostatic management of THA implantation for treating FNF. Postoperative haematoma formation is associated with various complications, with PJI being a major complication after THA [7,8,9]. A recently published study by Mortazavi et al. [8] showed that haematoma formation was an independent risk factor for poor outcomes. Affected patients had a lower Harris Hip Score, a lower Satisfaction Score, a greater complication rate, and a higher mortality following primary THA. In the study conducted by Galat et al., it was observed that reoperation for haematoma was significantly associated with an increased risk of PJI of the hip [37]. Therefore, achieving adequate surgical haemostasis reduces the need for blood transfusions and prevents complications such as heterotopic ossification. Further, it enhances haemodynamics, facilitates postoperative anticoagulation, minimises pain, and contributes to overall patient recovery [38]. 

Effective management of bleeding and the associated haemostasis is therefore crucial, particularly in the substantial patient population with coagulopathies resulting from the use of oral anticoagulants. The ideal haemostatic agent is biodegradable, has a rapid onset of action, and does not induce pathological thrombosis [39]. When selecting a haemostat for surgical use, considerations should include the accessibility of the bleeding site, the size of the wound, and the type of agent required. Surgeons must consider the intensity of the bleeding and factors such as the patient’s coagulation status and their medical or surgical history when selecting the most suitable haemostatic agent and evaluating the risk of bleeding. In orthopaedic surgery, haemostats and sealants constitute the primary categories of haemostatic adjuvant agents. Mechanical haemostats excel in managing minor bleeding by establishing a physical barrier to the blood flow and fostering a matrix that expedites swift clot formation. The common mechanical agents used include porcine gelatine, bovine collagen, oxidised regenerated cellulose, polysaccharide spheres, bone wax, and bone wax alternatives [31,40]. 

These haemostatic agents are often used in combination with thrombin. Notably, they are categorised within the same class as MPSHs, such as Arista^®^ AH [31,40]. Mechanical haemostatic agents operate by creating a physical barrier to stop bleeding, thereby aiding in natural coagulation [39,40]. They are regarded some of the safest and most cost-effective options for haemostasis in this domain. However, there is a notable consistent risk associated with these agents due to the potential swelling of the materials, attributed to their absorptive properties, as documented in studies [39,41]. While topical haemostatics have become widely utilised in current medical practice, their use and effects have been less extensively researched compared to TXA.

In surgical practices, the implementation of minimally invasive approaches, combined with hypotensive anaesthesia, electrocoagulative haemostasis, and the use of antifibrinolytics, has been highly effective in minimising blood loss. TXA is a frequently utilised agent, particularly in arthroplasty surgery, consistently proving its efficacy in preventing substantial haematoma formation and rare adverse systemic or local side effects [24,26]. In patients undergoing primary THA, intravenous, topical, and combined methods of TXA administration have all demonstrated significant reductions in both blood loss and the risk of transfusion when compared to a placebo [42]. There is no method of TXA administration shown to be clearly superior over the others for patients undergoing primary THA. In this regard, studies evaluating TXA in orthopaedic surgery show that it is effective and safe when administered both intravenously or intraarticularly [24,42]. The TXA mechanism works via reversible binding to the lysine receptor sites on plasminogen and by inhibiting the formation of plasmin, it results in increased antifibrinolytic effect and decreased bleeding. TXA has been proposed as potentially reducing the infection risk via a number of mechanisms, such as a reduction in transfusions and its effect on plasmin-mediated immune-modulating pathways. Further, haematoma formation provides a fertile growth medium for pathogens. There are nowadays a lot of studies confirming that TXA results in a reduction in PJI after joint replacement [43,44]. As an option for TXA delivery into the joint, direct injection of 3 g of TXA after fascial closure stands out as an option for TXA delivery into the joint as a targeted approach to enhancing its efficacy [45]. While TXA is generally deemed to be safe for the vast majority of patients, its use is contraindicated for those with a history of pulmonary embolism, venous thromboembolism, stroke, cardiac stents, cardiac bypass, or pro-coagulation disorders, which restricts its usage in many cases [46]. Consequently, for this specific patient category, the topical administration of haemostatic agents may present a safer alternative, potentially reducing the risk of thromboembolic complications. In our study, we used TXA as an intravenous and topical agent in both groups as the standard procedure for all patients. 

Absorbable haemostatic agents have become integral components in different surgical procedures, playing a pivotal role in the effective management of intraoperative bleeding and haemostasis. We introduced the usage of MPHSs into our practice over the last two years and assessed its effect in patients scheduled for THA due to FNF. Polysaccharide hemospheres are plant-derived polysaccharide starches utilised as mechanical haemostatic agents in surgical settings. An initial description of these haemostatic agents in an experimental setting was provided by Murat et al. in an animal study. In this research, they demonstrated that MPSHs offer rapid, effective, and durable haemostasis during partial nephrectomy in an animal model [47]. These agents act as sieves to dehydrate the blood, thereby increasing the concentration of coagulation factors and platelets to promote clotting [31,39,40]. Coagulation factors aggregate as they are absorbed by the polysaccharide hemospheres. Upon contact, clotting begins, achieving haemostasis within minutes. These haemostatic agents are available in powder form and are applied directly to the bleeding site. They possess an intermediate cost profile in comparison to other haemostatic agents. Demonstrating efficacy in reducing capillary, venous, and arteriolar bleeding, these agents are also noted for their absorption within 48 h post-application [30,39,48]. 

In our procedure, the agent was strategically placed locally within the hip joint prior to the closure of the fascia. The potential for soft tissue swelling is a recognised aspect of the action mechanism of MPSH technology [48]. The impact of this swelling becomes particularly significant in regions with delicate skin flaps. Studies conducted by Offodile et al. and Miller et al. have substantiated this phenomenon, reporting instances of distal tip skin flap necrosis and challenges in wound healing associated with the use of MPSHs [32,49]. However, in our investigation, despite the hip joint being encased in a robust layer of soft tissue, including muscles, capsules, fascia, and fat, we observed no excessive swelling that could be linked to the application of the studied substance. In our study, the routine application of MPSHs in total hip arthroplasty procedures was observed to be safe, with no local or systemic adverse events related to the product.

The utilisation of MPSHs is well documented across a range of animal models [47,49,50,51]. These studies involve non-coagulopathic animal models and draw comparisons between MPSHs and other less potent haemostatic agents, such as oxidised cellulose and collagen pads. In a prospective, multicentre, randomised, controlled clinical study, MPSHs were noninferior to a collagen haemostatic pad [51]. Further, they provided rapid, effective, and durable local haemostasis in a porcine open partial nephrectomy model [47]. ASHPs have been studied in various specialties, including cardiothoracic surgery, urology, general surgery, otolaryngology, and gynaecology [29,30,52,53]. M. Humphreys et al. evaluated microporous polysaccharide hemospheres, with demonstrated efficiency in achieving topical haemostasis in the setting of intracorporeal laparoscopic splenic injury [54]. Antisdel et al. have conducted several studies in the field of otolaryngology, revealing a consistent pattern of reduced early postoperative bleeding following endoscopic sinus surgery [52]. Notably, their research indicates no adverse effects on postoperative healing within the sinus cavity. Bruckner et al. detailed the use of Arista^®^ AH in the context of cardiothoracic surgery, demonstrating a notable reduction in the time required to achieve haemostasis and a decrease in the postoperative chest tube output [29]. Importantly, these positive outcomes were achieved without a corresponding increase in complications. Additionally, data on MPSH powder application in surgery for brain tumours showed improved local haemostasis [53]. At the same time, in another study by K. M. Lewis, which studied the haemostatic efficacy of microporous hemospheres compared to a heparinised porcine abrasion model of a capsular tear in a parenchymal organ, they were shown to have low-level efficacy, and it was suggested that MPSHs were not an appropriate surrogate for haemostatics [30]. Considering the available literature on MPSHs, which primarily focuses on their efficacy in general surgery procedures, the data consistently demonstrate a significant impact on achieving haemostasis.

While Arista^®^ AH has been extensively studied in the specialties mentioned above, its effectiveness in orthopaedic surgery, particularly in patients undergoing THA, remains an area of ongoing research. Limited studies have presented results regarding the application of studied starch in patients following hip arthroplasty. In a study conducted by Liu Tiansheng et al., the impact of 1 g of Arista^®^ AH was investigated in comparison to a control group comprising 98 patients who underwent unilateral total hip arthroplasty due to femoral neck fracture [33]. The results revealed notable advantages in the test group, as the total blood loss, drainage volume, and transfusion rate were significantly lower than those observed in the control group. Simultaneously, there were no discernible differences in the D-dimer levels, platelet count, activated partial thromboplastin time, and international normalised ratio between the two groups [33]. Studies assessing the use of MPSHs in THA have shown their potential benefits, including lower postoperative wound drainage, reduced haemoglobin levels, and diminished need for blood transfusions for osteoarthritis and fracture management [33,34]. 

In their study, Gleason et al. [35] observed that the topical application of absorbable surgical haemostatic powder did not significantly impact haematoma formation or wound infection rates in primary total knee arthroplasty for knee arthritis. They reported no substantial differences between the study groups, noting a tendency for more haematomas, infections, and increased transfusion rates in the Arista^®^ AH group. Similarly, our study did not reveal any significant outcomes, though we noted an overall positive trend in the analysed parameters when using MPSHs in hip surgeries. Our findings align with those of Gleason et al. [35]. However, we propose that this starch-based haemostat may be more suitably allocated for use in larger-volume surgeries, such as revisions, which are characterised by increased blood loss and a higher incidence of postoperative joint haematoma formation. They may also offer benefits for patients with coagulopathy or those on consistent anticoagulant therapy due to their heightened risk of bleeding. Nonetheless, further research is required to substantiate these suggestions. However, a correlation between haematoma formation and increased infection rates was observed, which underlines the need for precise haemostasis [35]. Sufficient haemostasis is crucial to reduce the risk of PJI in arthroplasty procedures. 

Our investigation showed a trend towards reduced bleeding complications in the Arista^®^ AH group, as well as lower incidences of PJI at the three-month follow-up. Nonetheless, these differences were not statistically significant in our analysis. In contrast to other studies, where the benefits of absorbable surgical haemostatic powder were more pronounced, a potential explanation for this could be the smaller wound areas and the minimally invasive surgical techniques employed for our cohort. Significant benefits of Arista^®^ AH in major thoracic and urological surgeries, which typically involve larger wound surfaces, has been previously documented in the literature. Consequently, the application of MPSHs may be particularly advantageous in revision surgeries, including arthroplasty revisions, where there is generally a higher incidence of blood loss and postoperative intra-articular haematoma formation [55]. This could be important for enhancing patient outcomes and reducing the risk of complications associated with PJI. 

Our investigation highlights the need for further research with a larger patient population to gather more comprehensive data on applying MPSH technology in orthopaedics. This study contributes to the orthopaedic field, shedding light on the effectiveness of MPSHs in patients after THA in FNF. However, further studies are needed to comprehensively understand the mechanism of action, efficacy, safety, and potential advantages of Arista^®^ AH and similar haemostatic agents in orthopaedic surgery.

This study has certain limitations, including the inherent biases associated with retrospective data analysis and chart reviews. Additionally, the study’s limited power might contribute to the absence of statistically significant differences in haematoma formation between groups. Although the follow-up period of three months is relatively short, we believe that it represents the most critical timeframe for assessing early periprosthetic joint infections (PJI) following total hip arthroplasty (THA). It is important to note that tranexamic acid was routinely used by surgeons at our institution during the study period.

On the other hand, the study has notable strengths. It was conducted in a single institution by two surgeons, effectively minimising confounding variables. The use of two consecutive time periods for the control and treatment groups reduces selection bias, as there were no specific patient selection criteria for the use of MPSHs. The patient demographics, combined with our carefully defined inclusion and exclusion criteria, contribute to a homogeneous and generalisable study population. Despite the relatively limited number of participants and statistical power, it can be said that this study stands as the most extensive investigation to date, offering valuable insights into the application of this absorbable haemostat in total hip arthroplasty for femoral neck fracture. Our preliminary results offer a valuable dataset for the field. This study holds more direct relevance to clinical practice in the orthopaedics specialty compared to animal studies exploring the use of this agent. However, further research is necessary to fully elucidate the mechanism of action, efficacy, safety, and potential benefits of Arista^®^ AH and similar haemostatic agents in orthopaedic surgery.

## 5. Conclusions

Our study confirmed the safety of routinely using Arista^®^ AH in patients undergoing THA implantation for FNF treatment. We observed no local soft tissue or systemic AEs associated with the use of MPSHs. There was no significant difference in the haemoglobin concentration and haematocrit and platelet values after the surgery between studied groups. Although there was a trend towards a reduced risk of bleeding complications, local haematoma formation, and cases with early PJI in the study group of patients, this trend did not reach statistical significance. Future research is necessary to elucidate the mechanism of action of Arista^®^ AH and explore further indications for using this haemostatic agent.

## Data Availability

The data presented in this study are available on request from the corresponding author. The data are not publicly available due to local institutional ethics board regulations.
